# Per-oral cross-facial sural nerve graft for facial reanimation

**DOI:** 10.1186/s40902-018-0163-3

**Published:** 2018-09-07

**Authors:** Joohee Jeong, Akram Abdo Almansoori, Hyun-Soo Park, Soo-Hwan Byun, Seung-Ki Min, Han-Wool Choung, Joo Yong Park, Sung Weon Choi, Bongju Kim, Soung-Min Kim, Jong-Ho Lee

**Affiliations:** 1 Department of Oral and Maxillofacial Surgery, School of Dentistry, Seoul National University, 275-1 Yeongeon-dong, Jongro-gu, Seoul, 110-749 South Korea; 2 Oral Oncology Clinic, Research Institute and Hospital, National Cancer Center, Goyang, South Korea; 3 Oral Cancer Center & Clinical Trial Center, Seoul National University Dental Hospital, Seoul, South Korea; 4 Dental Research Institute, Seoul National University, Seoul, South Korea; 5 Department of Oral and Maxillofacial Surgery, Dongtan Sacred Heart Hospital, Hallym University Medical Center, Kyonggi-do, South Korea; 6 Dental Life Science Research Institute, Clinical Translational Research Center for Dental Science, Seoul National University Dental Hospital, Seoul, South Korea

**Keywords:** Facial nerve paralysis, Cross-facial nerve graft, Facial reanimation, Sural nerve

## Abstract

**Background:**

Cross-facial nerve graft is considered the treatment of choice for facial reanimation in patients with unilateral facial palsy caused by central facial nerve damage. In most cases, a traditional parotidectomy skin incision is used to locate the buccal and zygomatic branches of the facial nerve.

**Methods:**

In this study, cross-facial nerve graft with the sural nerve was planned for three patients with facial palsy through an intraoral approach.

**Results:**

An incision was made on the buccal cheek mucosa, and the dissection was performed to locate the buccal branch of the facial nerve. The parotid papillae and parotid duct were used as anatomic landmarks to locate the buccal branch.

**Conclusions:**

The intraoral approach is more advantageous than the conventional extraoral approach because of clear anatomic marker (parotid papilla), invisible postoperative scar, reduced tissue damage from dissection, and reduced operating time.

## Background

Cross-facial nerve graft is considered the treatment of choice for facial reanimation in patients with unilateral facial palsy caused by damage to the proximal stump of the facial nerve. This procedure involves connecting the facial nerve branches of the unaffected side to the contralateral branches of the affected side or the nerves of grafted muscle for reanimation using a free nerve graft. Conventionally, a parotidectomy skin incision is made, and dissection is performed to locate the midfacial branches of the facial nerve, especially the buccal branch. There are two approach methods for finding the buccal branch: anterior and posterior approaches. Both methods, however, tend to leave a postoperative scar on the skin. Moreover, there are no clear anatomic markers for locating the nerve branches in the anterior approach, and the posterior approach carries a greater risk of damaging the trunk of the facial nerve. In this study, a cross-facial nerve graft using the sural nerve was performed intraorally on three patients with unilateral facial palsy.

## Methods

The first patient is a 52-year-old female, first noticed with a right facial palsy 2.5 years ago. She was diagnosed with pontomedullary glioma and received radiation treatment on the brain stem 1.5 years ago. The second patient was a 62-year-old female with Bell’s palsy. Paralysis had persisted for 50 years at the time of visit, and she had exophthalmos and ptosis on the right side. The third patient was a 54-year-old female who exhibited a left side facial palsy after meningioma removal 4 years previously. For all patients, clinical tests including needle electromyography and nerve conduction test were performed. And per-oral cross-facial sural nerve graft for facial reanimation was planned.

## Results

### Technical report

**Fig. 1:**
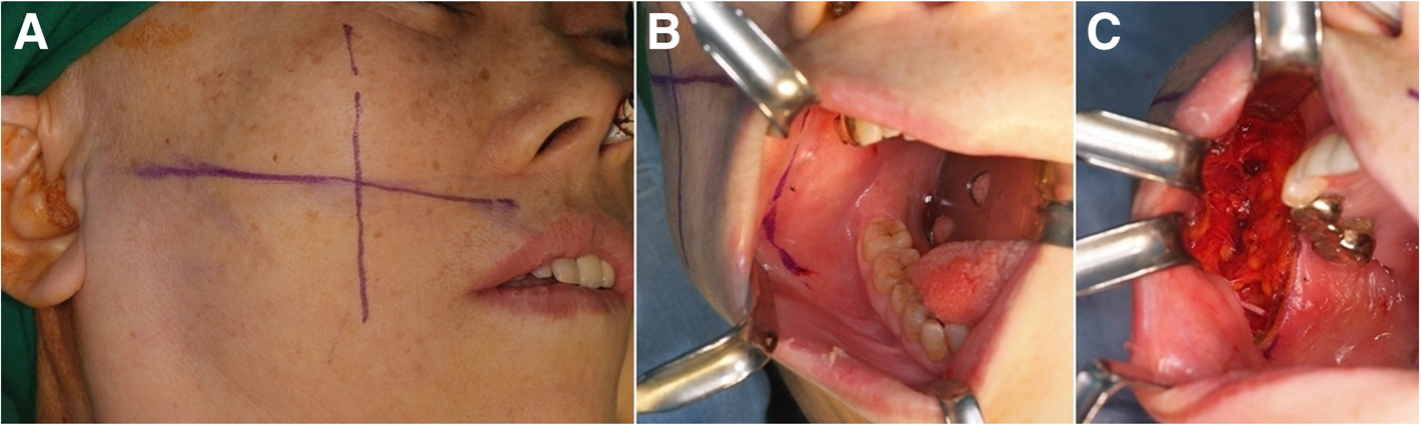
Clinical photographs showing the facial reanimation technique using per-oral cross-facial sural nerve graft. **a** Crossed lines were first drawn on the face to identify the closest point between the parotid duct and the buccal branch. **b** A 4-cm vertical incision line was drawn on the cheek buccal mucosa approximately 5 mm anterior to the parotid papilla. **c** Dissection was performed through the buccinator muscle, buccal fat pad, and posteriorly along the parotid duct to identify the buccal branch

**Fig. 2:**
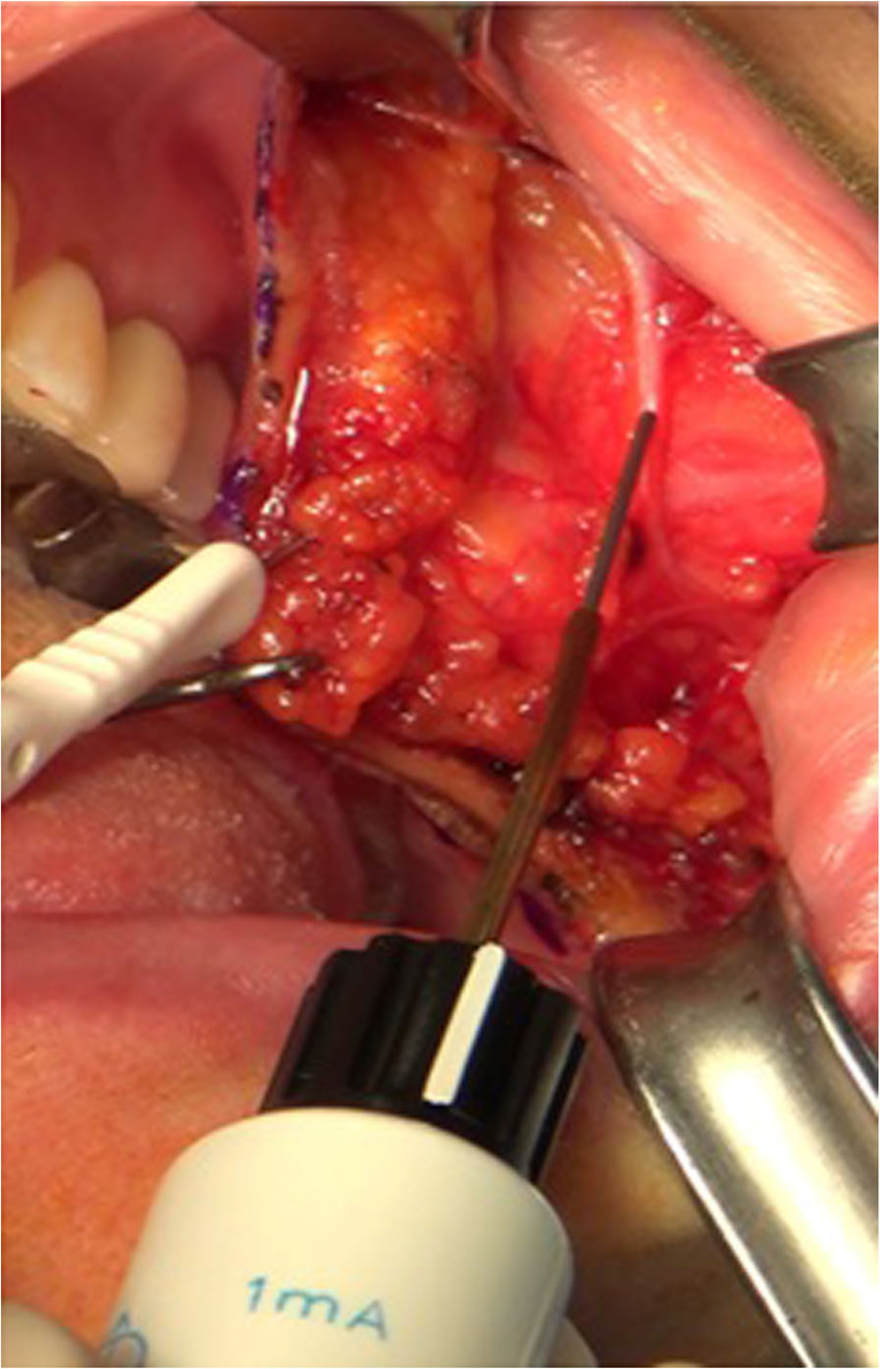
Identifying the buccal branch near the parotid duct using a nerve stimulator

**Fig. 3:**
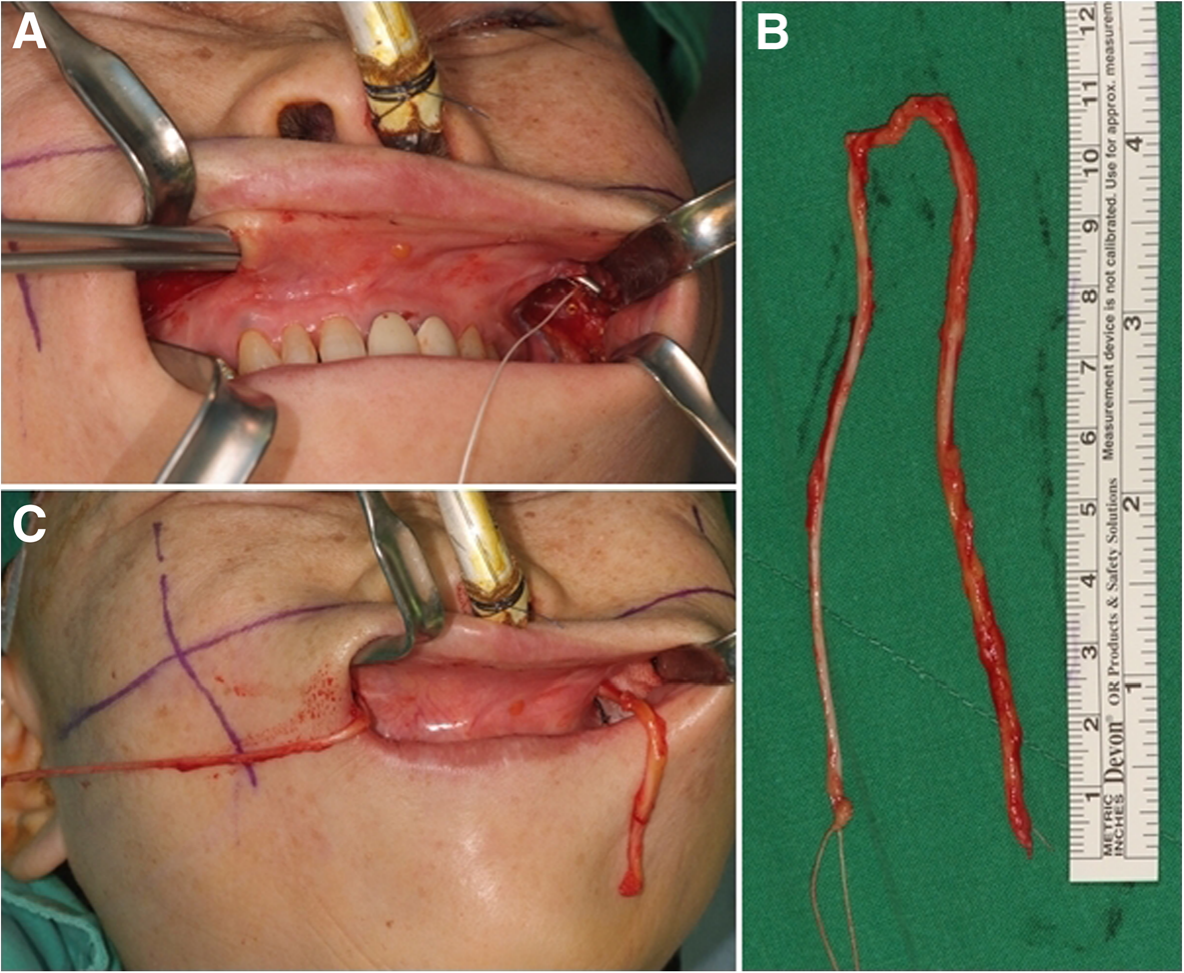
**a** A submucous tunneling made through the upper vestibular area from the left operating field to the right operating field for passing a harvested sural nerve. **b** The harvested sural nerve measured about 20 cm in length. **c** Passage of the harvested sural nerve through the submucosal tunnel

**Fig. 4:**
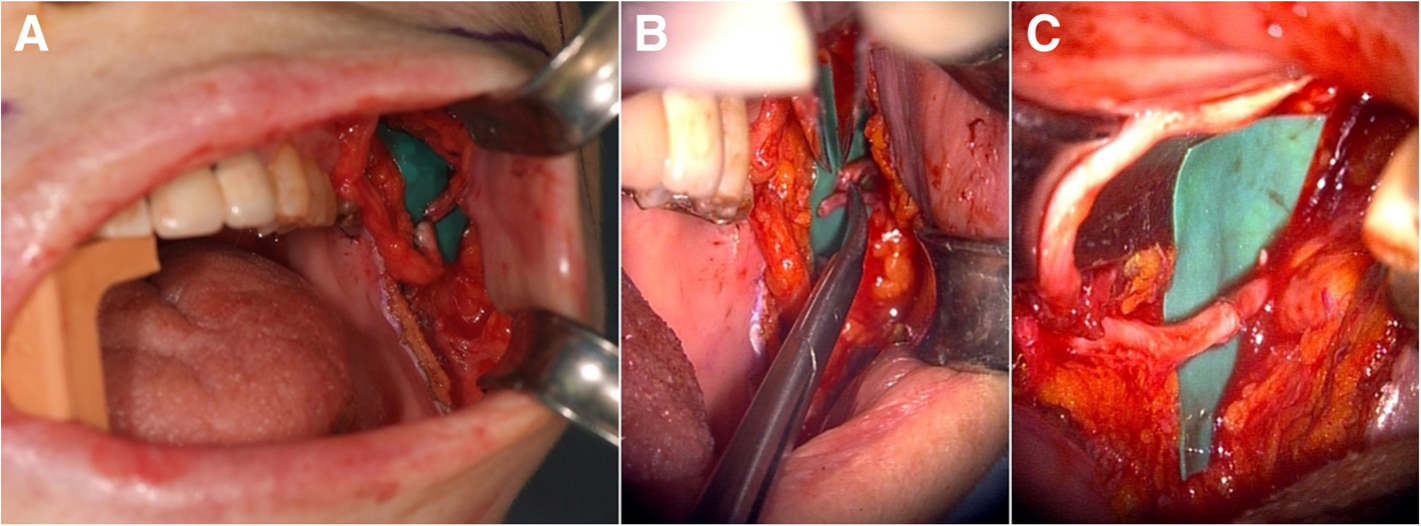
Clinical photographs showing the anastomosis of the sural nerve with the buccal branches of the facial nerve under a microscope. **a** Positioning of the harvested sural nerve with its distal end facing the non-paralyzed buccal branch. **b** Preparation of the buccal branch. **c** Placement of epineural sutures between the sural and buccal branch of the facial nerve in the non-paralyzed site

The predicted path of the facial nerves was drawn on the face using a surgical marking pen. A horizontal line was drawn from the center of the upper lip to the intertragic notch along the predicted path of the buccal branch of the facial nerve. A line was drawn past the anterior border of the masseter muscle and perpendicularly intersected the horizontal line (Fig. 1a). We estimated that the parotid duct maintained a parallel course with the buccal branch of the facial nerve to the cross point, after which it curved around the masseter muscle to enter the oral cavity [1]. The parotid papilla of the normal side was tagged with a 6-0 nylon stitch. A vertical incision of about 4 cm in length was made on the buccal mucosa 5 mm anterior to the parotid papilla (Fig. 1b). Access was prepared by incising the mucosa and submucosa. Further dissection was made through the buccinator muscle, buccal fat pad, and continued posteriorly along the parotid duct. A facial nerve branch that ran inferiorly along the parotid duct was located in the anterior margin of the masseter muscle. The buccal and zygomatic branches were carefully dissected and identified between the SMAS layer and the deep fascia (Fig. 1c). The buccal branches were confirmed using a nerve stimulator (Fig. 2). The same procedure was performed on the affected side. Both operating fields were connected through submucosal tunneling across the upper labial vestibule. The sural nerve (up to 20 cm in length) was harvested using a 2-cm-long incision on the postero-superior area of the lateral malleolus. The distal end of the harvested sural nerve was placed on the normal side and the proximal portion on the affected side through the submucosal tunnel (Fig. 3). The buccal branch of the non-affected facial nerve was transected and anastomosed with the sural nerve in an end-to-end fashion under a microscope (Fig. 4). On the affected side, the sural nerve ending was divided into two fascicles and anastomosed to the buccal branch of the facial nerve using an end-to-end technique and to the zygomatic branch using an end-to-side technique. When a cross-facial nerve graft was planned for future facial reanimation using free muscle graft, the affected side was not prepared. Instead, the upper labial vestibular and subcutaneous tunnel to the affected preauricular area was made with medium Kelly, and the proximal end of the sural nerve was introduced to this area after the distal end of the sural nerve was anastomosed to the non-affected buccal branch for secondary surgery.

## Discussion

Since Scaramella’s report in 1971, cross-facial nerve graft has been considered the treatment of choice for patients with facial nerve damage at the proximal stump level [2, 3]. Traditionally, long skin incisions and wide dissections were required for skin flap elevation and to locate specific branches of the facial nerves in this technique. In our cases, however, a cross-facial nerve graft was successfully performed via an intraoral mucosal incision and dissection without wide elevation of the facial skin. This per-oral approach was advantageous over the conventional extraoral approaches for several reasons.

First, the presence of a stable anatomic landmark, the parotid papilla, aids in locating the parotid duct, which is closely associated with the course of the facial nerve’s buccal branch. There have been numerous studies on anatomic markers associated with the paths of the facial nerves and their branches. The facial nerve divides into five branches within the parotid gland, and the buccal branch courses along the parotid duct between superficial and deep fascia in the masseter muscle region. Pogrel et al. reported that the distance between the parotid duct and the buccal branch of the facial nerve measured approximately 5.43 ± 3.65 mm [1]. Son et al. reported that the distance was about 2.54 ± 1.48 mm [4]. The parotid duct arises from the anterior aspect of the parotid gland and passes anteriorly in the masseter muscle region with a close proximity to the buccal branch of the facial nerve. At the anterior border of the masseter muscle, the duct turns medially through the buccal fat pad and buccinator muscle to its associated oral mucosa papillae [5, 6]. It is therefore possible, with sufficient anatomic knowledge and clinical experience, to locate the buccal branch of the facial nerve through intraoral incision and dissection along the parotid duct from the parotid papilla.

Second, the invasive extraoral approach that consists of skin flap elevation and wide dissection to the anterior border of the parotid gland is unnecessary. Wilhelmi et al. reported that the mean distance between the anterior border of the parotid gland and tragus was 38.8 mm [7]. Therefore, a flap over 40 mm in length needs to be elevated from the preauricular incision line to find the buccal branch. However, with an intraoral approach, the range of dissection and depth of the elevated flap were no more than 20 mm from the incision line made just anterior to the parotid papilla. In addition to reduced morbidity from a smaller incision and less dissection, scar on the facial skin can also be avoided through the intraoral approach.

Finally, the intraoral approach required a shorter operation time. As mentioned above, because of a reduced dissection and smaller flap size, along with relatively convenient upper vestibular tunneling, the procedure allows for an easier, faster surgery. The presence of reliable anatomical structures—the parotid papillae and parotid duct—also helps reduce operation time.

## Conclusions

We performed three cross-facial nerve grafting procedures using the intraoral approach. Although this technique is novel, it is highly advantageous for both physicians and patients. Additional cases and procedures should be assessed to optimize the per-oral technique before it can be substituted for the extraoral approach.

## Abbreviation

SMAS: Superficial muscular aponeurotic system
